# Immature platelets: A useful tool for thrombocytopenia etiology classification in adults

**DOI:** 10.1016/j.htct.2026.106469

**Published:** 2026-05-21

**Authors:** Raphael Ferreira Pimentel, Tayná Regina Leal dos Santos, Marcos André Cavalcanti Bezerra, Betânia Lucena Domingues Hatzlhofer, Maria Tereza Cartaxo Muniz

**Affiliations:** a University of Pernambuco, Brazil; b Hematology and Hemotherapy Foundation of Pernambuco, Brazil; c Federal University of Pernambuco, Brazil

**Keywords:** Immature platelet fraction, Reticulated platelets, Thrombocytopenia, Laboratory automation

## Abstract

**Introduction:**

The etiology of thrombocytopenia is diverse with heterogenous clinical manifestations which demand the correct diagnosis and appropriate management. The immature platelet fraction is associated with new platelet release from the bone marrow by thrombopoiesis. The clinical utility of this low-cost, feasible measurement has been demonstrated across various conditions. This study aimed to assess the immature platelet fraction and count as diagnostic tests to distinguish between the two main causes of thrombocytopenia in selected hematology disorders.

**Methods:**

A total of 444 participants were evaluated: Group 1 (n = 179; healthy blood donors), Group 2 (n = 131; peripheral platelet destruction or consumption, including immune thrombocytopenia and thrombotic thrombocytopenic purpura), and Group 3 (n = 134; bone marrow failure secondary to aplastic anemia, chemotherapy, acute leukemia, or multiple myeloma).

**Results:**

Median immature platelet fraction (%) and immature platelet count (× 10^9^/L) values were 3.3/8.1 (Group 1), 16.5/6.1 (Group 2), and 5.0/1.8 (Group 3), with significant intergroup differences between Groups 2 and 3 (p-value <0.001). Optimal diagnostic cut-offs were 8.3% for immature platelet fraction (AUC = 0.8585) and 3.1 × 10^9^/L for immature platelet count (AUC = 0.7687). The immature platelet fraction was highest in immune thrombocytopenia patients and showed significant inverse correlations with platelet counts in Groups 1 and 2 (p-value <0.001).

**Conclusion:**

Immature platelet fraction and immature platelet count can be useful to discriminate between the main etiologies and should be included in the routine of hematology centers for screening thrombocytopenia.

## Introduction

Thrombocytopenia is a clinical condition defined by a platelet count (PLT) below 150 × 10^9^/L. Since platelets are responsible for primary hemostasis and the maintenance of the integrity of the blood vessel walls, platelet counts lower than 20 × 10^9^/L represent a tendency of spontaneous bleeding. Despite the intensity of the thrombocytopenia, the clinical heterogeneity observed in patients is elevated and the bleeding tolerance varies according to its etiology. The main mechanisms for a low PLT are increased destruction or consumption of platelets and decreased production. Typical examples of the latter are the bone marrow (BM) failure syndromes (e.g., aplastic anemia [AA], myelodysplastic syndromes [MDS], and chemotherapy-induced thrombocytopenia), or marrow-infiltrative processes (e.g., acute leukemia [AL], multiple myeloma [MM]). Increased destruction or consumption, on the other hand, is seen in conditions such as immune thrombocytopenia (ITP) and the thrombotic microangiopathies (TMA) [1, 2, 3].

Thrombocytopenia management is tailored to the care setting, typically categorized by outpatient, inpatient, or emergency department requirements [2]. In addition, an understanding of the underlying pathophysiological processes involved in thrombocytopenia and the differential diagnoses is key to appropriate management. Platelet transfusions, for example, are not helpful for ITP yet they remain a mainstay of prophylactic care in AA [1,4].

Establishing the cause of thrombocytopenia has obvious clinical repercussions but is sometimes quite challenging. Beyond the clinical history and physical examination, the laboratory plays a critical role in the diagnostic workup. This includes an extensive investigation of biomarkers for coagulation screening, biochemical profiles, and peripheral blood smear examination. If the etiology of thrombocytopenia is unclear, a BM aspirate and biopsy should be performed to preclude a primary BM disorder [2]. Quantification of plasma glycocalicin and thrombopoietin levels via enzyme-linked immunosorbent assay (ELISA) can be helpful to assess platelet turnover [5]. Platelet indices have also been useful for this assessment but are not standardized and their analysis is affected by storage time [6]. However, ‘young platelets’ have been reported to be an effective biomarker for distinguishing between increased destruction or consumption of platelets and decreased production [7, 8, 9, 10].

Initially identified as reticulated platelets and quantified via flow cytometry, these cells were difficult to adopt in routine practice due to significant technical challenges and a lack of international standardization [11,12]. Advances in fluorescence-based platelet detection on fully automated hematology analyzers have made it possible to quantify the fraction of RNA-rich immature platelets, known as the immature platelet fraction (IPF). This parameter is now available as part of a routine complete blood count (CBC), providing rapid results in approximately one minute at a low cost. It is analogous to the reticulocytes observed in erythropoiesis due to recent release of platelets into the bloodstream from the BM and represents the rate of thrombopoiesis, that is, a surrogate marker for the assessment of BM function [8].

Elevated IPF levels have been reported in cases of thrombocytopenia secondary to increased peripheral destruction, reflecting a compensatory BM response characterized by accelerated thrombopoiesis. Diminished IPF has also been found in cases of ‘hypoproductive’ thrombocytopenia [9,10]. This study assessed the IPF and immature platelet count (IPC) in selected hematological disorders to establish their clinical utility in the diagnosis of patients with thrombocytopenia and contribute to the standardization of these parameters in the differentiation of two main etiologies.

## Method

### Study design

This cross-sectional diagnostic accuracy study was developed in the Hematology and Hemotherapy Foundation of Pernambuco, northeastern Brazil from June 2023 to July 2024. The study population consisted of three adult cohorts: healthy blood donors (Group 1, n = 179); patients with peripheral platelet destruction or consumption (Group 2, n = 131), including various clinical phases of ITP and acute thrombotic thrombocytopenic purpura (TTP); and patients with impaired platelet production (Group 3, n = 134), encompassing AA, MM, and AL at diagnosis or during chemotherapy.

Inclusion criteria were defined as: (i) adults aged >18 years; (ii) patients with a platelet count <150 × 10^9^/L; (iii) a confirmed diagnosis of ITP, TTP, AL, AA, or MM; (iv) patients with acute myeloid leukemia on day 14 or 15 post-induction therapy with daunorubicin and cytarabine (DA 3+7); and (v) healthy blood donors with a normal CBC. Exclusion criteria were the presence of conditions which cause thrombocytopenia such as infection, liver diseases, folate or vitamin B12 deficiency and pseudo thrombocytopenia observed by the microscope evaluation of blood smears.

This study commenced after the approval of the local Ethics and Research Committee (project registration number CAAE 67609023.4.0000.5195) and approval (number 6.077.121). The blood samples of the patients were part of the routine diagnostic workup. All patients with AL and TTP came from the emergency department, while most of the patients with ITP and MM came from the outpatient clinic and all chemotherapy patients came from the hematology ward. All blood donors and patients who were invited for the research signed an informed consent form.

### Sample collection

Samples of 4 mL of peripheral blood were obtained through venipuncture using vacuum tubes containing ethylenediaminetetraacetic acid (EDTA) K3 (Cral, São Paulo, Brazil). Blood samples were gently inverted to ensure homogenization, stored at room temperature, and transported for analysis within 12 h.

### Measurement of immature platelet fraction and other variables

The PLT was performed in a Sysmex XN-1000 full-automated hematology analyzer, (Sysmex Corporation, Kobe, Japan) employing the fluorescent method with a dedicated channel for this purpose. Fluorescence flow cytometry utilizes an oxazine-based fluorescent dye and a semiconductor laser to identify and quantify platelets. The dye penetrates the cell membrane to stain nucleic acids within the rough endoplasmic reticulum and mitochondria, allowing for the differentiation of immature platelets based on their higher fluorescence intensity. Two-dimensional scattergrams were plotted based on the collected data, with forward scattered light (FSC) and side fluorescent light (SFL) representing the Y-axis/cell volume and X-axis/cytoplasmic RNA, respectively. Thus, the IPF is determined by an algorithm which discriminates the larger platelets with intense RNA content from mature platelets. The IPC was calculated by multiplying the IPF by the PLT and dividing by 100. White blood cells (WBC) and hemoglobin were measured in the same analyzer. To assure the performance of the analyzer, three commercial internal quality control samples (Sysmex e-Check) were run before beginning the laboratory routine. Demographic data (age and sex) were obtained from electronic medical records for patients and electronic blood bank records of blood donors.

## Statistical analysis

Data were analyzed using the STATA SE-64 statistical software package (Stata Corp LP, Texas, USA). The normality of the quantitative variables was assessed using the Shapiro-Wilk’s test. As the distribution of PLT, IPF, and IPC deviated from normality across all groups, these variables are expressed as medians and interquartile ranges (IQR). Differences of these variables between the groups were evaluated by the Kruskal-Wallis and Dunn’s post hoc tests and then expressed as box plots. Receiver operating characteristic (ROC) curves were constructed to determine the cut-off value for IPF and IPC to differentiate Group 2 from Group 3 with the area under curve (AUC) being calculated with a 95% confidence interval (95% CI). Correlation analyses between PLT and IPF for the three groups used the Spearman correlation test and are expressed as scatter plots. A p-value ≤0.05 was considered statistically significant in all analyses.

## Results

A total of 444 participants were evaluated in this study. Of these, 265 had thrombocytopenia and were allocated to groups according to their diagnosis: elevated destruction/consumption of platelets (Group 2) and reduced platelet production (Group 3). A predominance of female participants was observed in Group 2, whereas the highest median age was recorded in Group 3. The median IPF of Group 1 was 3.3% (Range: 0.6%−14.1%). Demographic characteristics of the three groups are summarized in Table 1. The analysis based on the patient diagnosis shows that the median IPF was highest in ITP (16.5%) and lowest in AA. Regarding Group 3, the highest median IPF was in AL (6.4%), followed by MM (4.6%), AA (3.4%) and chemotherapy (1.5%). Data of the immature platelet parameters are shown in Table 2.

**Table 1:** Descriptive analysis of healthy individuals and patient groups.

	Group 1 (n = 179)	Group 2 (n = 131)	Group 3 (n = 134)
Age (years) – median (range)	34 (27–46)	45 (28–57)	52.5 (35–68)
Male – n (%)	125 (69.8)	29 (22.1)	59 (44.0)
Female – n (%)	54 (30.2)	102 (77.9)	75 (56.0)
Hemoglobin (g/dL) - median (IQR)	14.8 (13.7–15.7)	13.5 (12.3–14.4)	8.2 (7.1–9.6)
WBC (x10^9^/L) - median (IQR)	7.0 (6.0–8.2)	8.1 (6.6–10.6)	5.4 (2.3–17.1)
PLT (x10^9^/L) - median (IQR)	256 (230–293)	38 (14–89)	40 (17–78)
		39 (15.0–81.0) *	
IPF (%) - median (IQR)	3.3 (2.2–4.7)	16.5 (9.7–27.1)	5.0 (2.8–9.0)
		9.2 (4.4–18.4) *	
IPC (x10^9^/L) - median (IQR)	8.1 (5.9–11.8)	6.2 (2.9–9.9)	1.8 (0.7–3.8)
		3.4 (1.3–7.6) *	

PLT: Platelet Count; WBC: (White blood cells; IPF: Immature platelet fraction; IPC: Immature platelet count; IQR: interquartile range.

⁎ Measurements were performed on all thrombocytopenic patients, regardless of underlying etiology.

**Table 2:** Platelet parameters of patient groups according to the blood disorders.

	Group 2 (n = 131)		Group 3 (n = 134)			
	ITP (n = 122)	TTP (n = 9)	AL (n = 84)	AA (n = 25)	AML + chemotherapy (n = 8)	MM (n = 17)
PLT (x10^9^/L)	41 (16–93)	14 (9–17)	31 (11.5–60)	47 (23–116)	22 (14.5–44)	82 (66–122)
IPF (%)	16.9 (10.5–28)	9 (6.8–18.1)	6.4 (3.3–12)	3.4 (2.6–9.2)	1.5 (1.0–2.0)	4.6 (2.8–7.3)
IPC (x10^9^/L)	6.5 (3.5–9.8)	1.2 (0.6–2.3)	1.7 (0.7–3.3)	2 (1.2–3.4)	0.4 (0.2–0.8)	4.2 (2.3–5.2)

PLT: Platelet Count; IPF: Immature platelet fraction; IPC: Immature platelet count; ITP: Immune thrombocytopenia; TTP: Thrombotic thrombocytopenic purpura; AA: Aplastic anemia; AL: Acute leukemia; AML Acute myeloid leukemia; MM: Multiple myeloma.

Data expressed as median and interquartile range (IQR).

**Figure 1:**
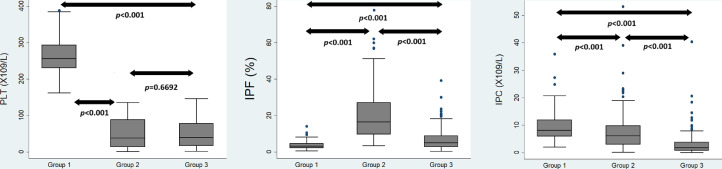
Comparison of platelet count (PLT) and immature platelet parameters across the study cohorts: Group 1 (healthy controls), Group 2 (thrombocytopenia secondary to increased destruction or consumption), and Group 3 (thrombocytopenia secondary to bone marrow failure). IPF: Immature platelet fraction; IPC: Immature platelet count.

There was no significant difference between PLT in the thrombocytopenic groups. Patients in Group 2 had significantly higher median IPF compared to the other groups. Additionally, the IPC was significantly different between the groups (Figure 1).

**Figure 2:**
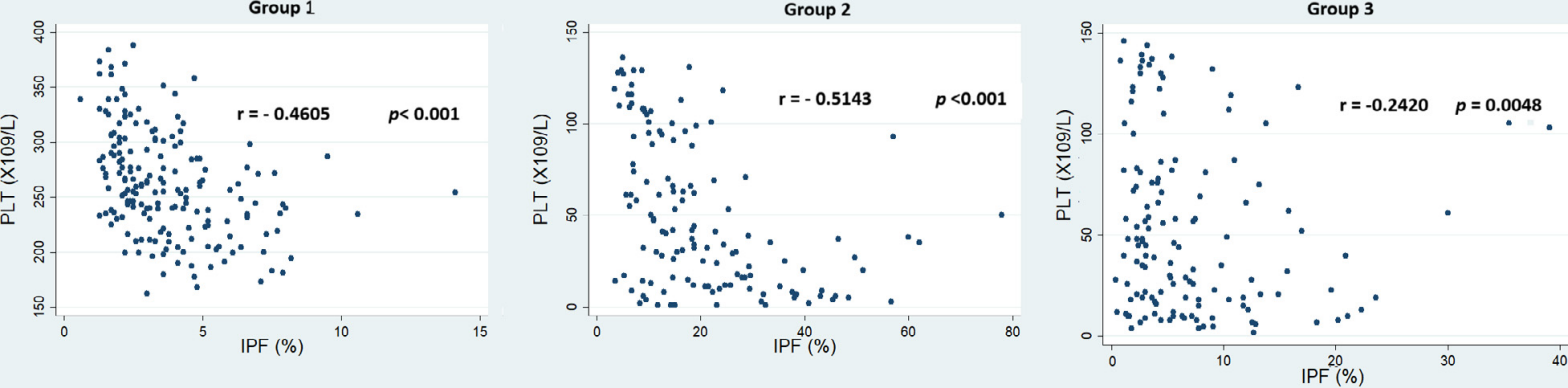
Distribution of the immature platelet fraction (IPF%) in relation to the platelet count (PLT) across the three study groups: Group 1 (healthy individuals); Group 2 (thrombocytopenia secondary to increased platelet destruction or consumption); and Group 3 (thrombocytopenia secondary to bone marrow failure).

The immature platelet fraction in thrombocytopenic patients was nearly 300% higher than in healthy subjects. This finding does not indicate that the decrease in the PLT always correlates with an increase in immature platelets. Figure 2 illustrates an inverse correlation between IPF and PLT across all groups. This relationship was moderate in Groups 1 and 2, whereas Group 3 exhibited a significantly weaker association.

**Figure 3:**
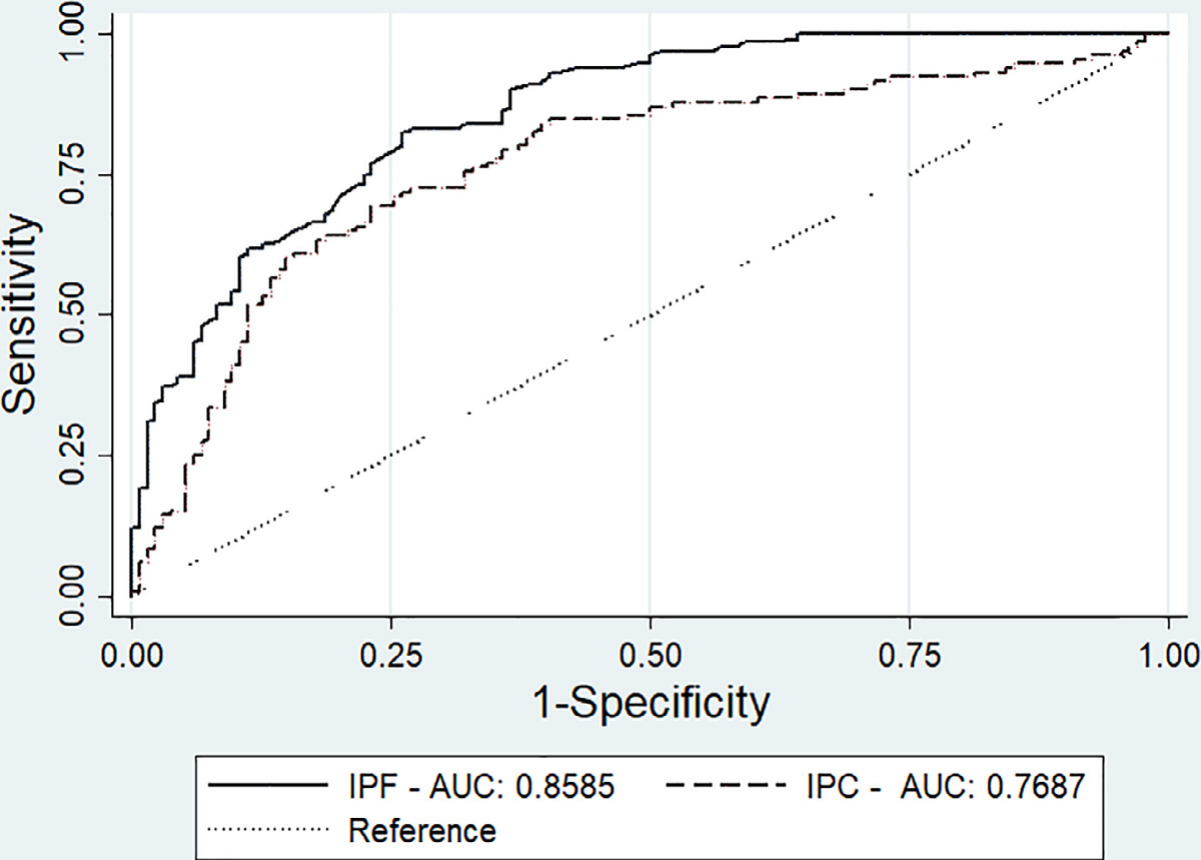
Receiver operating characteristics (ROC) curves and areas under the curve for immature platelets. IPF: Immature platelet fraction; IPC: Immature platelet count.

The diagnostic accuracy of the IPF and IPC measurements for the etiological distinction of the thrombocytopenia was estimated using ROC analysis (Figure 3). The AUC for the differentiation between Groups 2 and 3 were 0.8585 (95% CI: 0.8153–0.9017; p-value <0.001) for IPF and 0.7687 (95% CI: 0.7101–0.8273; p-value *<*0.001) for IPC. Both parameters were statistically higher in cases of increased destruction or consumption of platelets than in diminished production of platelets. The optimal IPF and IPC values for discriminating between the groups were determined using the ROC curve. For IPF, a cut-off set at 8.3% yielded a sensitivity of 83.2% and a specificity of 73.1%. For IPC, a cut-off set at 3.1 × 10^9^/L yielded a sensitivity of 72.5% and a specificity of 67.9%.

## Discussion

Immature platelets are newly produced platelets from BM with a high RNA content with its quantity mirroring thrombopoiesis. These platelets were photographed by electron microscopy over a dozen years ago; they have been shown to have longevity and to be highly biosynthetically active [13]. In clinical conditions characterized by increased platelet turnover and megakaryocyte activity, the IPF can be rapidly quantified via fully automated hematology analyzers. This parameter is measured simultaneously with the standard CBC.

Over the last twenty years, a great number of studies have been developed demonstrating the change in the IPF in different clinical conditions including thrombocytopenia [8].

In Group 1, the median IPF was comparable to the values reported by Ali et al. [14]; despite this cohort being significantly smaller than theirs (n = 2292) and not exclusively limited to blood donors for healthy subject selection, both studies utilized analyzers from the same manufacturer. Furthermore, the same methodology for outlier removal was employed utilizing standard CBC reference ranges. Imperiali et al. followed the Clinical and Laboratory Standard Institute (CLSI) guidelines to enroll a normal group with a similar sample size (n = 153) and mean PLT and found that the median IPF and IPC were comparable with the present study [15]. A lower median IPF and IPC were observed in another study with a smaller sample size (n = 105) carried out using a different series of the analyzer within 30 min of sample collection [9]. Although the current study analyzed blood samples stored at room temperature for a maximum of 12 h, no significant variation had been reported for the stability of assays during 48 h of observation in previous studies [7,16].

Given the high frequency of ITP patients in Group 2, the findings of this study can be compared with research that only included ITP patients. Similar to the present study, the median IPF in these studies was significantly higher than in other clinical groups [9,17,18]. ITP is characterized by immune mechanisms that boost platelet destruction thereby shortening platelet lifespan, consequently causing the percentage of immature platelets to increase. The diagnosis is still reached by the exclusion of other causes of thrombocytopenia, a process that carries a considerable risk of misdiagnosis [19]. Some previous diagnostic criteria warranted revision, such as the lack of specificity of platelet-associated IgG and the absence of specific findings in the BM examination [20]. Since there is no gold standard laboratory marker for this disorder, IPF is potentially useful to differentiate ITP from hereditary macrothrombocytopenia [21] and AA [9,18]. Recent studies proposed that, despite the limited usefulness of IPF alone, its combination with other laboratory evidence can significantly improve the accuracy of the diagnosis of ITP from other causes of thrombocytopenia [20,22]. As for the acute TTP subjects in Group 2, the increased IPF and decreased PLT compared to Group 1 illustrate the higher consumption of platelets due to pathogenic mechanisms. This parameter might have an important role in patient monitoring during plasmapheresis since a previous study observed a decreased IPF in TTP after remission [7]. Kier et al. described an IPF value which guided the therapy in a single thrombotic microangiopathic case, but this study demands prospective clinical trials to validate the finding [23].

Group 3 comprised diverse hematological disorders sharing a common etiology of central thrombocytopenia, albeit with differing underlying mechanisms of BM failure. Diminished platelet production caused by BM infiltration due to hematological malignances such as AL and MM showed median IPFs above that of the healthy subjects. Ali et al. evaluated this parameter using a different analyzer series from the same manufacturer in a larger cohort that included lymphoma, chronic leukemia, and AL. Their reported median IPF of approximately 5.0% is in agreement with the findings of the present study. However, another study that included patients with lymphoblastic and acute myeloid leukemia found a higher IPF (around 10.0%) [17]. In accordance with the present study, all previously reported research demonstrated a significantly higher IPF compared to control groups. Malignant precursor cell infiltration is a dynamic process in which these cells exert a proliferative advantage over other lineages, ultimately leading to bone marrow suppression and subsequent thrombocytopenia [24]. The variation in increased immature platelets may be explained by the progressive infiltration of the bone marrow by malignant blasts or plasma cells. In this model, thrombopoiesis is proportionally impaired as healthy tissue is displaced; however, further correlation studies between IPF and blast percentages are warranted to confirm this relationship. Prospective studies of immature platelets in malignant disorders have been performed by monitoring platelet recovery after chemotherapy and BM transplantation [7,9]. Regarding the clinical utility of this parameter in hematological malignancies, a recent study utilized artificial neural network predictive modeling to demonstrate that the IPF, alongside two other analyzer-derived parameters, may effectively flag cases of acute promyelocytic leukemia [25].

The lower medians of IPF and IPC (approximately three times less) observed in Group 3 compared with Group 2 illustrate the impaired thrombopoiesis which results in thrombocytopenia. This finding agrees with Ashraf et al. who also studied ITP, TTP, AA and AL subjects in their research evaluating IPF [26]. The median IPF was similar to the results of Goel et al. (4.6%) [10] but higher than those of Jeon et al. (1.8%) [22]. This result is expected since 85.7% of the patients in Group 3 in the latter study were cancer patients undergoing chemotherapy; previous studies show that chemotherapy patients have substantially lower IPF values [7,27]. Thrombocytopenia secondary to chemotherapy is a frequent hematologic complication of myelosuppressive cancer therapy the main mechanisms of which are the inhibition of platelet-derived growth factor and apoptosis of megakaryocytes [28].

In the present study, an inverse correlation between IPF and PLT was observed across all groups; however, this association was not statistically robust in Group 3. This lack of significance may be attributed to the subset of AL patients, who exhibited the highest median IPF within that cohort, consistent with previous reports. The correlation in Group 2 was also observed by Ferreira et al. (r = −0.70) whose patient population included ITP patients [21]. Hence, the increase in IPF is related to the reduced PLT in Groups 1 and 2.

Regarding diagnostic performance, the IPF demonstrated high discriminatory power in distinguishing Group 2 from Group 3. The findings of this study are consistent with the AUC values reported by Goel et al. (0.8680), Jeon et al. (0.9310), and Sakuragi et al. (0.9560) [9,10,18].

The IPC for the control group observed here had a similar range compared to previous studies. While IPC demonstrated good discriminatory power between groups, its diagnostic accuracy was slightly inferior to that of the IPF, a finding that aligns with prior research [9]. In terms of clinical utility, the IPC may be preferable to the IPF in scenarios where the fraction does not accurately reflect the physiological state. This includes determining the appropriateness of platelet transfusions in thrombocytopenic patients at high risk of bleeding, as well as monitoring treatment response in ITP subjects [29,30].

Despite rigorous selection criteria aimed at excluding external influences, such as infectious or nutritional factors, and recruiting subjects from a specialized hematology and hemotherapy center, this study has limitations. Specifically, the sample sizes of the chemotherapy and TTP subgroups were small. Furthermore, the clinical stage of disease and the specific treatment regimens were not investigated. As both IPF and IPC reflect active thrombopoiesis, the potential influence of these variables cannot be entirely ruled out.

## Conclusions

This study demonstrates that IPF and IPC are substantially lower in patients with BM failure compared with those with conditions involving peripheral platelet destruction and consumption. Thus, the clinical utility of immature platelets for differentiating thrombocytopenia due to distinct pathogenesis with acceptable accuracy in agreement with previous studies involving adults is evident. Since this test is cheap and automated, it should be a standard part of initial blood work for patients with low platelet counts.

## Ethics of approval statement

All procedures performed in studies involving human participants were in accordance with the ethical standards of the national research committee.

## Patient consent statement

Informed consent was obtained from all invited participants included in the study.

## Permission to reproduce material from other sources

For this type of study consent for publication is not required.

## Clinical trial registration

For this type of study clinical trial registration is not required**.**

## Author contributions

Raphael Ferreira Pimentel conceived and designed the study, recruited patients, updated the clinical data, performed experiments, performed statistical analyses, analyzed and interpreted data, and drafted the manuscript. Tayná Regina Leal dos Santos performed experiments, recruited patients and updated the clinical data. Marcos André Cavalcanti Bezerra, Betânia Lucena Domingues Hatzlhofer and Maria Tereza Cartaxo Muniz, analyzed and interpreted data and reviewed the manuscript. Maria Tereza Cartaxo Muniz gave the final approval of the version to be submitted.

## Conflicts of interest

The authors declare that they have no conflict of interest.

## Data Availability

The data that support the findings of this study are available from the corresponding author upon reasonable request.

## References

[1] Izak M., Bussel J.B. (2014). Management of thrombocytopenia. F1000Prime Rep.

[2] Stasi R. (2012). How to approach thrombocytopenia. Hematology Soc Hematol Educ Program.

[3] Santoshi R.K., Patel R., Patel N.S., Bansro V., Chhabra G. (2022). A comprehensive review of thrombocytopenia with a spotlight on intensive care patients. Cureus.

[4] Townsley D.M., Desmond R., Dunbar C.E., Young N.S. (2013). Pathophysiology and management of thrombocytopenia in bone marrow failure: possible clinical applications of TPO receptor agonists in aplastic anemia and myelodysplastic syndromes. Int J Hematol.

[5] Bodrova V.V., Shustova O.N., Khaspekova S.G., Mazurov A.V. (2023). Laboratory markers of platelet production and turnover. Biochemistry (Moscow).

[6] Vinholt P.J., Hvas A.M., Nybo M. (2014). An overview of platelet indices and methods for evaluating platelet function in thrombocytopenic patients. Eur J Haematol.

[7] Briggs C., Kunka S., Hart D., Oguni S., Machin S.J. (2004). Assessment of an immature platelet fraction (IPF) in peripheral thrombocytopenia. Br J Haematol.

[8] Buttarello M., Mezzapelle G., Freguglia F., Plebani M. (2020). Reticulated platelets and immature platelet fraction: clinical applications and method limitations. Int J Lab Hematol.

[9] Jeon K., Kim M., Lee J., Lee J.S., Kim H.S., Kang H.J. (2020). Immature platelet fraction: a useful marker for identifying the cause of thrombocytopenia and predicting platelet recovery. Medicine (United States).

[10] Goel G., Semwal S., Khare A., Joshi D., Amerneni C.K., Pakhare A. (2021). Immature platelet fraction: its clinical utility in thrombocytopenia patients. J Lab Physicians.

[11] Robinson M., Machin S., Mackie I., Harrison P. (2000). In vivo biotinylation studies: specificity of labelling of reticulated platelets by thiazole orange and mepacrine. Br J Haematol.

[12] Kienast J., Schmitz G. (1990). Flow cytometric analysis of thiazole orange uptake by platelets: a diagnostic aid in the evaluation of thrombocytopenic disorders. Blood.

[13] Hille L., Lenz M., Vlachos A., Grüning B., Hein L., Neumann F.J. (2020). Ultrastructural, transcriptional, and functional differences between human reticulated and non-reticulated platelets. Journal of Thrombosis and Haemostasis.

[14] Ali U., Knight G., Gibbs R., Tsitsikas D.A. (2017). Reference intervals for absolute and percentage immature platelet fraction using the Sysmex XN-10 automated haematology analyser in a UK population. Scand J Clin Lab Invest.

[15] Imperiali C.E., Arbiol-Roca A., Sanchez-Navarro L., Dastis-Arias M., Lopez-Delgado J.C., Cortes-Bosch A. (2018). Reference interval for immature platelet fraction on sysmex xn haematology analyser in adult population. Biochem Med (Zagreb).

[16] Noel M.M., Feugray G., Kasonga F., Buchonnet G., Daliphard S., Barbay V. (2023). Stability over time of immature platelet fraction and comparison between EDTA and citrated whole blood samples. J Clin Lab Anal.

[17] Butt A.J., Zaidi U., Munawar Ali R., Zafar S., Ali M.S., Shamsi T. (2023). Reticulated platelet count as a diagnostic tool in immune thrombocytopenia (ITP). Cureus.

[18] Sakuragi M., Hayashi S., Maruyama M., Kabutomori O., Kiyokawa T., Nagamine K. (2015). Clinical significance of IPF% or RP% measurement in distinguishing primary immune thrombocytopenia from aplastic thrombocytopenic disorders. Int J Hematol.

[19] Bussel J., Cooper N., Boccia R., Zaja F., Newland A. (2021). Immune thrombocytopenia. Expert Rev Hematol.

[20] Kashiwagi H., Kuwana M., Murata M., Shimada N., Takafuta T., Yamanouchi J. (2024). Reference guide for the diagnosis of adult primary immune thrombocytopenia, 2023 edition. Int J Hematol.

[21] Ferreira F.L.B., Colella M.P., Medina S.S., Costa-Lima C., Fiusa M.M.L., Costa L.N.G. (2017). Evaluation of the immature platelet fraction contribute to the differential diagnosis of hereditary, immune and other acquired thrombocytopenias. Sci Rep.

[22] Jeon M.J., Yu E.S., Kang K.W., Lee B.H., Park Y., Lee S.R. (2020). Immature platelet fraction based diagnostic predictive scoring model for immune thrombocytopenia. Korean J Intern Med.

[23] Kier Y.E., Stempak L.M., Maitta R.W. (2013). Immature platelet fraction can help adjust therapy in refractory thrombotic microangiopathic hemolytic anemia cases. Transfusion and Apheresis Science.

[24] Nachtkamp K., Kobbe G., Gattermann N., Germing U. (2023). New methods of diagnosis, prognostication, and treatment. Dtsch Arztebl Int.

[25] Haider R.Z., Ujjan I.U., Shamsi T.S. (2020). Cell population data–Driven acute promyelocytic leukemia flagging through artificial neural network predictive modeling. Transl Oncol.

[26] Ashraf S., Rehman S., Asgher Z., Hamid A., Qamar S. (2020). Comparison of immature platelet fraction (IPF) in patients with central thrombocytopenia and peripheral thrombocytopenia. J Coll Physicians Surg Pak.

[27] Jung H., Jeon H.K., Kim H.J., Kim S.H. (2010). Immature platelet fraction: establishment of a reference interval and diagnostic measure for thrombocytopenia. Ann Lab Med.

[28] Hambardzumyan L., Grigoryan H., Badikyan M., Khachatryan H., Sargsyan N., Sulikhanyan A. (2023). Disparities in the consensus for treatment of chemotherapy-induced thrombocytopenia. ecancer.

[29] Bat T., Leitman S.F., Calvo K.R., Chauvet D., Dunbar C.E. (2013). Measurement of the absolute immature platelet number reflects marrow production and is not impacted by platelet transfusion. Transfusion (Paris).

[30] Ko Y.J., Hur M., Kim H., Choi S.G., Moon H.W., Yun Y.M. (2015). Reference interval for immature platelet fraction on Sysmex XN hematology analyzer: a comparison study with Sysmex XE-2100. Clin Chem Lab Med.

